# Polymer-Assisted Polymorph Transition in Melt-Processed Molecular Semiconductor Crystals

**DOI:** 10.1021/acs.chemmater.4c00418

**Published:** 2024-06-03

**Authors:** Pallavi Sundaram, Rochelle B. Spencer, Akash Tiwari, St. John Whittaker, Trinanjana Mandal, Yongfan Yang, Emma K. Holland, Christopher J. Kingsbury, Mia Klopfenstein, John E. Anthony, Bart Kahr, Sehee Jeong, Alexander G. Shtukenberg, Stephanie S. Lee

**Affiliations:** † Molecular Design Institute, Department of Chemistry, New York University, New York, New York 10003, United States; ‡ Department of Chemistry, University of Kentucky, Lexington, Kentucky 40506, United States; § Cambridge Crystallographic Data Centre (CCDC), 12 Union Road, Cambridge CB2 1EZ, U.K.

## Abstract

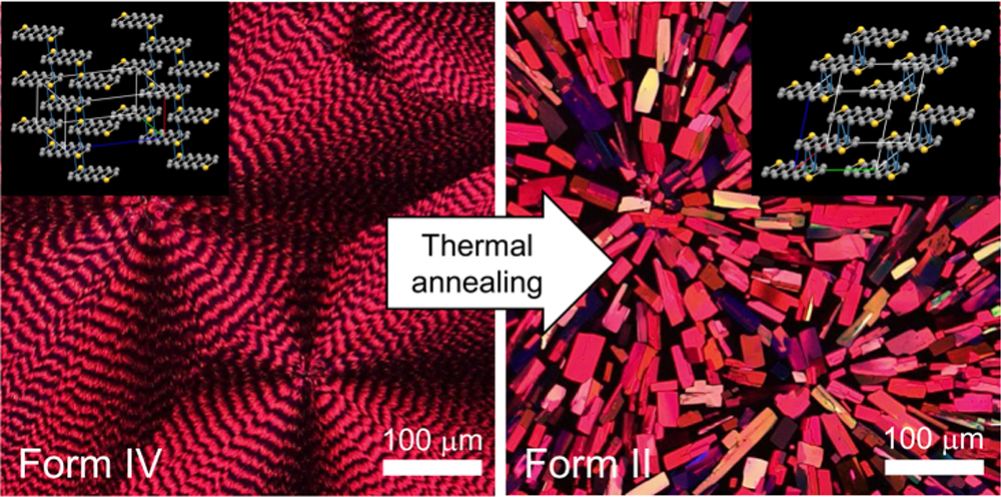

A previously unreported polymorph of 5,11-bis(triisopropylsilylethynyl)anthradithiophene (TIPS ADT), Form II, crystallizes from melt-processed TIPS ADT films blended with 16 ± 1 wt % medium density polyethylene (PE). TIPS ADT/PE blends that initially are crystallized from the melt produce twisted TIPS ADT crystals of a metastable polymorph (Form IV, space group *P*1̅) with a brickwork packing motif distinct from the slipstack packing by solution-processed TIPS ADT crystals (Form I, space group *P*2_1_/*c*) at room temperature. When these films were cooled to room temperature and subsequently annealed at 100 °C, near a PE melting temperature of 110 °C, Form II crystals nucleated and grew while consuming Form IV. The growth rate and orientations of Form II crystals were predetermined by the twisting pitch and growth direction of the original banded spherulites in the melt-processed films of the blends. Notably, the Form IV → II transition was not observed during thermal annealing of neat TIPS ADT films without PE. The presence of the mobile PE phase during thermal annealing of TIPS ADT/PE blend films increases the diffusion rate of TIPS ADT molecules, and the rate of nucleation of Form II. Form IV crystals are more conductive but less emissive compared to Form II crystals.

## Introduction

Advances in organic electronics have been made both by designing new molecules with a large potential overlap between π-molecular orbitals^1^ and by controlling their solid-state structures.^2^ Field-effect transistors comprising solution-processed films of lattice-strained triisopropylsilylethynyl (TIPS) pentacene crystals, for example, exhibit hole mobilities as large as 4.6 cm^2^/V·s, almost six times larger than those comprising films of unstrained TIPS pentacene crystals.^3^ Similarly, the mobilities of five different polymorphs of *n*-type organic semiconductors of two-dimensional quinoidal terthiophene vary over five orders of magnitude.^4^

In the chemical industry, systematic polymorph screening typically involves sublimation, solution-processing, postfabrication treatments, and growth under nanoconfinement.^5,6^ Recently, crystallization from the melt was proposed as a high throughput screening method for new polymorphs that form under large crystallization driving forces.^7^ In particular, melt crystallization under nanoconfinement revealed new polymorphs of carbamazepine, oxalyl dihydrazide, and sulfameter, which were not accessible via other crystallization methods.

**Scheme 1:**
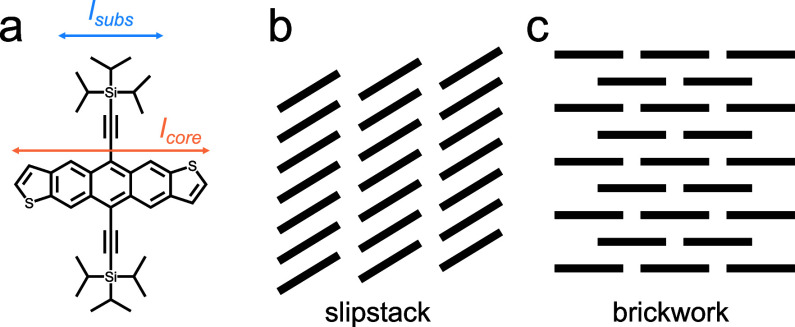
(a) TIPS ADT Molecular Structure; (b, c) Schematic of Slipstack and Brickwork Packings Adopted by TIPS ADT

We recently used melt crystallization to identify the structure of a previously unreported polymorph of 5,11-bis(triisopropylsilylethynyl) anthradithiophene (TIPS ADT),^8^ in which conjugated acene cores are functionalized with bulky solubilizing side groups. Solution-grown TIPS ADT crystals adopt a slipstack (*P*2_1_/*c*) packing, hereafter referred to as Form I, with molecules π-stacked in parallel columns, an arrangement generally observed for silyl-functionalized acenes in which the diameter of the silyl substituents, *l*_subs_, is more than half of the length of the conjugated core, *l*_core_ (Scheme 1a,b).^9^ TIPS ADT crystals adopt a brickwork *P*1̅ packing (Scheme 1c), hereafter referred to as Form IV, when crystallized from the melt instead.^8^ This structure is typically observed in functionalized acenes for which *l*_subs_ is approximately one-half *l*_core_ and leads to higher charge mobilities due to a two-dimensional network of π-orbital overlap between adjacent molecules.^9^ We discovered that Form IV crystallization proceeds through the nucleation and growth of spherulites of straight crystals and banded spherulites comprising helicoidal crystals^10^ in the absence and presence of 16 wt % medium density polyethylene (PE), respectively, directly from the melt.^9^ Form IV crystals persisted for months at room temperature, although some conversion to Form I was observed.

Here, we report the transformation of Form IV into two unreported polymorphs, named Form II and III. Form II crystallized in the presence of PE at 100 °C, while Form III formed spontaneously at room temperature in the absence of PE. Form II and III crystals are structurally similar to Form I and IV crystals, respectively. Form IV → II transition during sample annealing suggests that enhanced mobility of PE chains near the annealing temperature increases TIPS ADT diffusivity and promotes the nucleation and growth of Form II. Conductivity maps of partially annealed TIPS ADT films revealed that Form II single crystals exhibit significantly lower conductivity compared to polycrystalline Form IV spherulites. We attribute this lower conductivity to a less extensive π-orbital overlap network. Melt crystallization in the presence of polymers can be a viable strategy for polymorph discovery and highlights the interactions between small molecules and polymers in blended films.

## Results and Discussion

**Figure 1:**
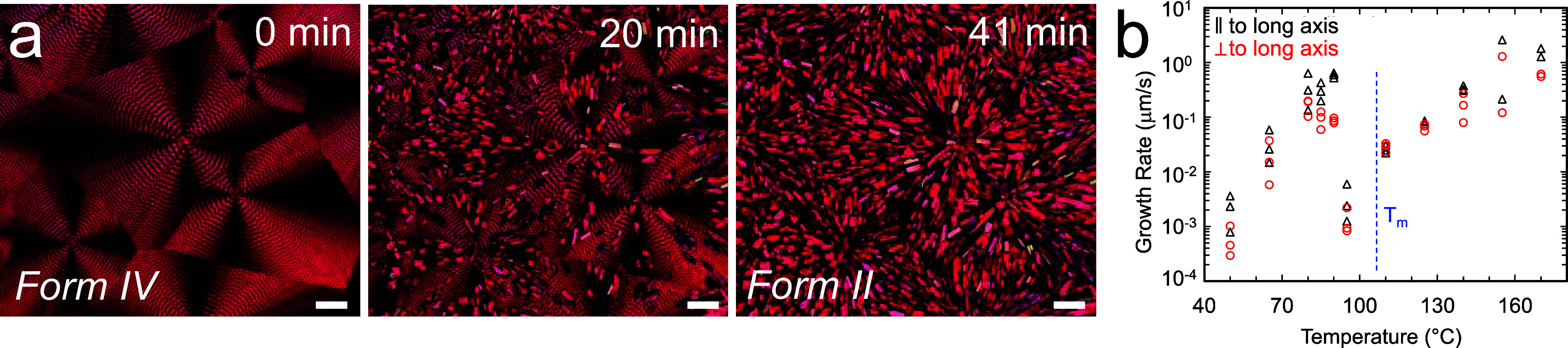
(a) Time-dependent images of a TIPS ADT banded spherulite film between crossed polarizers during thermal annealing at 100 °C. Scale bar = 100 μm. (b) Growth rate dependence on annealing temperature for Form II crystals. *T*_m_ indicates the melting point of medium density PE.

TIPS ADT thin films with 16 wt % medium density PE were crystallized from the melt between two glass slides following a previously reported procedure.^8^ When crystallized at 70 °C, TIPS ADT formed banded spherulites of twisted crystals emanating radially from a central nucleus, as displayed in the optical micrograph collected between crossed polarizers in Figure 1a (left). The average pitch, *P*, i.e., the spacing between like-colored concentric bands corresponding to 180° rotations of the out-of-plane orientation as lamellae twist about the growth direction, was 8 ± 0.5 μm. Melt-processed TIPS ADT adopts a high-temperature polymorph with a brickwork packing (Form IV), which is distinct from the slipstack packing of TIPS ADT crystals formed from solution at room temperature (Form I).^11^ Form IV was found to persist for at least several months in air at room temperature.

### Polymer-Assisted Polymorph Conversion

Following crystallization and cooling to room temperature, TIPS ADT Form IV films were thermally annealed at 100 °C for 60 min, a common technique in organic semiconductor thin film processing to increase crystallite size.^12−14^ This temperature is well below the TIPS ADT melting point of 207.7 °C but near the PE melting point of 106.7 °C (see Figure S1 for DSC curves). Figure 1a displays time-lapsed micrographs of the films between crossed polarizers during annealing (see also Video S1 and Figure S2 for brightfield images). Banded spherulites were replaced by rectangular single crystals with average lengths of 70 ± 10 μm that nucleated throughout the annealing process. Complete recrystallization took approximately 50 min. *In situ* X-ray diffraction collected during thermal annealing confirmed a polymorph transition from Form IV to a new polymorph, hereafter referred to as Form II, on a similar time scale (Figure S3).

For reference, a TIPS ADT film without PE was crystallized from the melt, cooled to room temperature, and thermally annealed at 100 °C. Form IV was stable throughout the annealing step (Figure S4), indicating that PE is necessary to induce the Form IV → II transition. In the absence of PE, Form IV transforms into the most stable Form I instead of Form II over months at room temperature. We also repeated thermal annealing experiments with both low density and high density PE with melting points of 103.2 and 122.4 °C, respectively (see Figure S5 for DSC curves). Time-lapse optical micrographs (Figure S6) and XRD patterns (Figure S7) confirmed that the Form IV → II conversion was largely insensitive to the PE molecular weight and crystallinity.

Figure 1b displays the linear growth rate of Form II crystals measured parallel (black triangles) and perpendicular (red circles) to the spherulite radial direction as a function of annealing temperature. Overall, the growth rate of Form II crystals was faster along the spherulite radial direction than perpendicular to the spherulite radial direction. Two distinct growth regimes were observed with the transition close to the PE *T*_m_ (indicated by blue dashed line in Figure 1b). Between 170 and 95 °C, well below the TIPS ADT Form IV melting point of 207.7 °C, the linear growth rate decreased exponentially with decreasing temperature. In this regime, we expect PE to act as a highly viscous solvent to provide enough mobility for TIPS ADT molecules to relax to the thermodynamically favored configuration but insufficient mobility for rearrangement into completely new microstructures. Form II growth can be arrested at any point in time by cooling the film to room temperature.

Surprisingly, lowering the temperature from 95 to 90 °C resulted in a two orders of magnitude increase in the linear growth rate. This dramatic increase in growth rate was accompanied by a shift in the Form II crystal morphology from single rectangular plates to irregular needles that preferentially nucleate at spherulite boundaries (Figure S8). The growth rate again decreased exponentially with decreasing temperature between 90 and 50 °C. We speculate that TIPS ADT local mass transport is facilitated along microcracks in the PE phase, which is a semicrystalline solid in this temperature range. A significant increase in TIPS ADT growth rate close to the melting point of the PE additive resembles a similar strong (up to three orders of magnitude) increase in growth rate of some molecular crystals close to glass transition temperature, *T*_g_.^15,16^ The mechanism of this so-called glass to crystal growth mode is not well understood, but the leading hypothesis assumes faster mass transport due to formation of microcracks in the material below the *T*_g_.^17,18^

Previously, polymers have been used to tune organic semiconductor diffusivity in films. Thermal annealing drives phase separation of TIPS pentacene and poly(α-methylstyrene) in blend films, resulting in higher quality crystalline domains of TIPS pentacene.^19^ Increasing polymer chain mobility via solvent vapor annealing induces reversible polymorphic transitions in molecular semiconductors.^20^ In TIPS ADT films, the Form IV → II transition rate is prohibitively small in the absence of mobile PE chains. Over months of storage at room temperature, Form IV converts directly to Form I both in the absence and presence of PE.

### Transformation Rate Dependence on Pitch

Increasing the crystallization temperature, *T*_c_, during initial formation of Form IV banded spherulites decreases the crystallization driving force, resulting in thicker crystalline fibrils that twist less aggressively than thinner fibrils.^21−26^ To examine the effect of pitch on Form IV → Form II conversion kinetics, we repeated annealing experiments with Form IV films crystallized at 130 °C, exhibiting a pitch of 90 ± 30 μm (see Figure S9 and Video S2). Hereafter, films crystallized at 70 and 130 °C will be referred to as *P*_8_ and *P*_90_, respectively, the subscript indicating pitch in microns.

**Figure 2:**
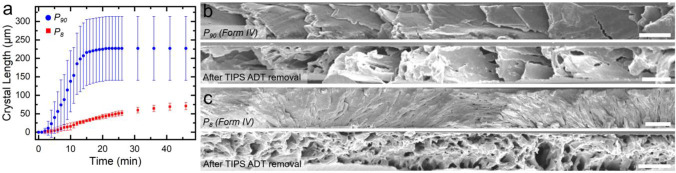
(a) Form II crystal size as a function of annealing time at 100 °C for TIPS ADT/MDPE *P*_90_ and *P*_8_ films. Error bars represent the standard deviation of crystal lengths for five different crystals measured at each time point in each film. TIPS ADT was selectively removed from the films by washing the film in acetone, which dissolves TIPS ADT but not PE. Scanning electron micrographs of cross sections of (b) *P*_90_ and (c) *P*_8_ films before and after the selective removal of TIPS ADT. These images were collected prior to thermal annealing at 100 °C. Scale bar = 2 μm.

Figure 2a displays the average lengths and standard deviations of five distinct crystals in *P*_8_ and *P*_90_ films during 50 min of thermal annealing at 100 °C. On average, crystals appeared within 10–15 s after annealing began (Videos S1 and S2). Form II crystal growth rates for the *P*_8_ and *P*_90_ films, extracted from the slope of the crystal size versus time in the linear portion of the curve, were 3 and 24 μm/min, respectively.

This 8-fold difference in conversion rates in *P*_8_ vs *P*_90_ films suggests that polyethylene chain mobility during thermal annealing is not the only factor affecting Form IV → Form II conversion. Figure 2b,c displays the cross-sectional scanning electron micrographs (SEMs) of *P*_90_ and *P*_8_ films before (top) and after (bottom) selective removal of TIPS ADT by dissolution in acetone. In the *P*_90_ film, platelike crystals with widths spanning the film thickness (*ca*. 3 μm) and lateral widths of ∼0.4 μm were observed. Fibrils adopted an edge-on orientation at the right side of the image and began to transition to a flat-on orientation toward the left side (Figure 2a). SEM images collected on PE after selective TIPS ADT removal revealed 1.6 ± 0.3 μm-wide cavities spanning the film thickness.

In contrast, lamellae observed in the cross-sectional SEM image of the *P*_8_ film had smaller widths and thicknesses compared to those in the *P*_90_ film, although insufficient contrast between individual crystallites hindered quantification. Both edge-on and face-on orientations were present within the scan window due to the small twisting pitch. After selective TIPS ADT removal, a nanoporous PE phase was observed, with 0.3 ± 0.1 μm cavities evenly distributed through the film. This cavity size, indicative of the TIPS ADT crystal size, is more than five times smaller than the average cavity size in the *P*_90_ films and is consistent with the general relationship of decreasing crystal thickness with decreasing pitch observed for other molecular compounds that form banded spherulites.^14,18^ The smaller extent of phase separation between TIPS ADT and PE in the *P*_*8*_ film compared to the *P*_90_ film is a consequence of the crystallization temperature during initial film formation. Nucleation and crystallization occur more rapidly with increasing undercooling, resulting in less time for TIPS ADT and PE to phase-separate.

Cross-sectional SEM images suggest that differences in Form II crystal morphologies, orientations, and growth rates between *P*_90_ and *P*_8_ films during thermal annealing are likely related to differences in the extent of phase separation between PE and TIPS ADT. In *P*_90_ films, TIPS ADT and PE phase-separate into 1–2 μm domains during initial crystallization from the melt, whereas phase separation occurs on the hundreds of nanometers length scale in *P*_8_ films. During recrystallization to Form II, TIPS ADT crystals in *P*_8_ films must displace PE to a larger extent compared to crystals in *P*_90_ films to form micron-sized single crystals. The physical barrier presented by PE chains when growing TIPS ADT Form II crystals in *P*_8_ films thus results in slower growth rates compared to *P*_90_ films. The stronger correlation between TIPS ADT crystal sizes and orientations before and after thermal annealing in *P*_90_ films compared to *P*_8_ films may also be related to the extent of phase separation. In *P*_8_ films, TIPS ADT must diffuse through the highly intermixed PE phase to incorporate into growing Form II crystals. In *P*_90_ films, on the other hand, TIPS ADT already formed large domains of pure Form IV crystals during initial film formation. TIPS ADT molecules do not need to diffuse over long distances during thermal annealing-induced recrystallization to Form II, thus retaining some “memory” of their orientation in the original twisted crystal film.

### Microstructural Analysis via Optical Polarimetry

Mueller matrix imaging (MMI)^27,28^ was used to analyze the microstructural details of TIPS ADT films before and after thermal annealing. Images were collected on a home-built petrographic microscope with two rotating quarter-wave plates above and below the sample, which form a complete polarization state generator.^29−31^ The transmitted light intensity was collected as a function of the continuously rotating quarter-wave plates. Digital demodulation was used to derive the 4 × 4 Mueller matrix, *M*, or polarization transfer matrix elements. *M* is given by:
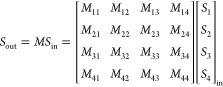
where *S*_in_ and *S*_out_ are the Stokes polarization states of incoming and exiting light, respectively, for any one pairwise configuration of the polarization state generator and polarization state analyzer. Optical properties can be extracted from the matrix *m*:
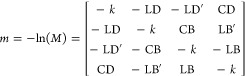
where *k* is the isotropic absorption, LD is the linear dichroism, LB is the linear birefringence, CD is the circular dichroism, and CB is the circular birefringence. LB’ and LD’ refer to differences measured for an intermediate reference frame with axes of ±45° with respect to the unprimed quantities.

The differential Mueller matrix *m* can be expressed as above if *M* is nondepolarizing. Linear and circular retardances and extinctions, LR, CR, LE, and CE, respectively, are thickness-dependent quantities associated with the intrinsic birefringence and dichroic ratios, LB, LD, CB, and CD.^28^

**Figure 3:**
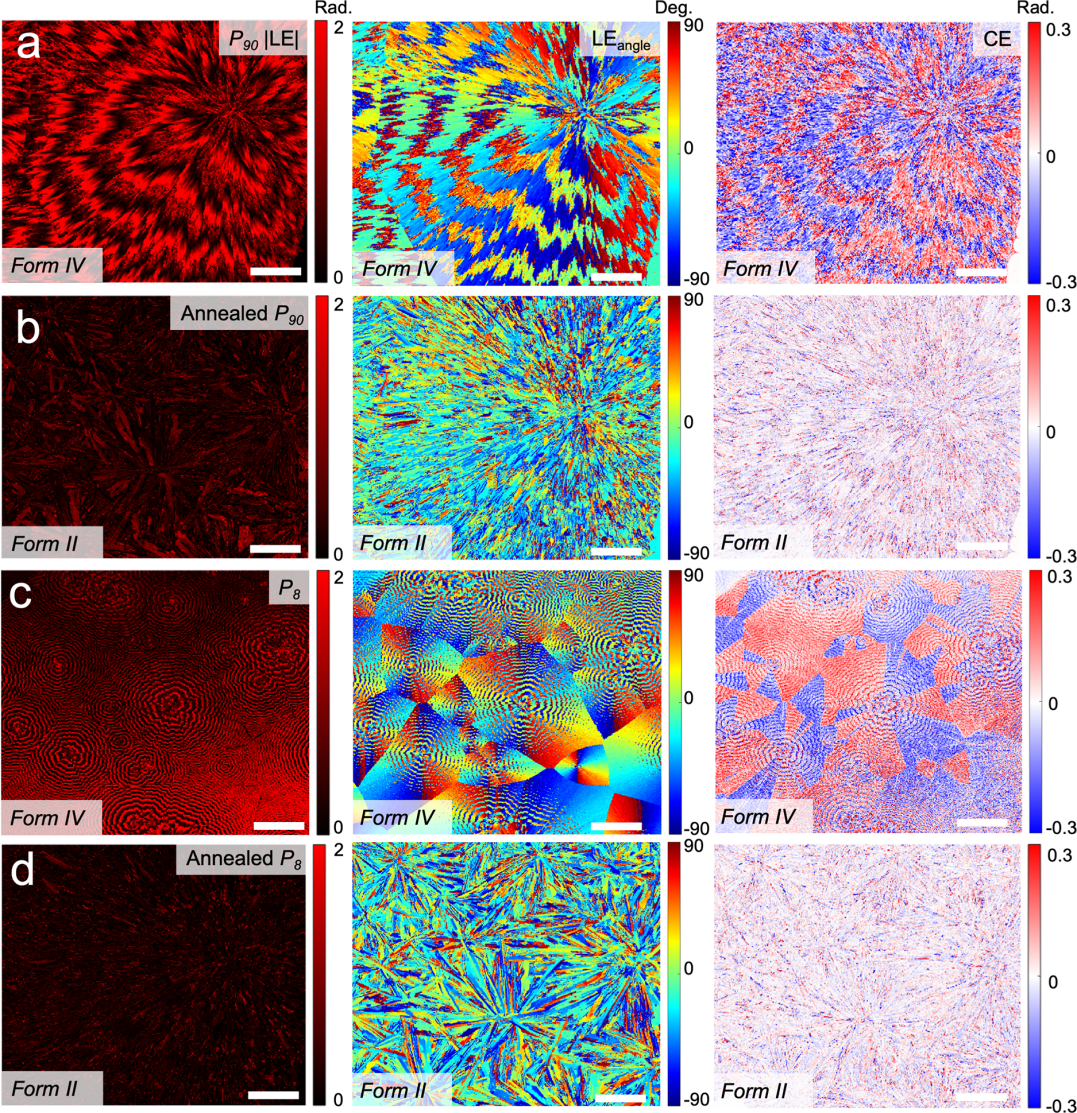
Linear extinction (LE) maps, angle-dependent linear extinction (LE_angle_) maps measured in degrees counterclockwise from the horizontal direction, and circular extinction (CE) maps of *P*_90_ and *P*_8_ films (a, c) before and (b, d) after annealing, respectively, collected at λ = 600 nm. Scale bar = 200 μm.

The row of panels indicated by Figure 3a displays the |LE|, LE_angle_ (the most absorbing polar direction plotted counterclockwise from the horizon), and CE maps of a *P*_90_ film, from left to right, respectively. In line with our previous findings, the LE signal of the *P*_90_ film oscillates along the spherulitic radial growth direction with the twisting bands, a consequence of the modulation of the extinction coefficients by continuous rotation of out-of-plane crystallographic orientations.^8,32^ The LE_angle_ map shows that crystals are oriented radially albeit the most absorbing direction alternates between radial and tangential as the crystals twist. Solid-state spectra previously collected reveal opposing polarization-angle absorption dependencies between the dark and bright bands at λ = 600 nm, corresponding to the π–π* transition.^8^ While dark bands, corresponding to the (001) plane oriented parallel to the substrate, exhibit strongest absorption when light is polarized along the radial direction, the opposite is true for light bands, corresponding to the (100) plane oriented parallel to the substrate.

Distinct dextrorotatory (red) and levorotatory (blue) domains are revealed in the CE map of the *P*_90_ film. It is not uncommon for centric crystals to grow in opposite directions with heterochiral properties, nor is it uncommon for the chiral properties to alternate sign along the direction of the crystal twist.^10,32−36^ TIPS ADT Form IV adopts a centrosymmetric crystal structure (space group *P*1̅) that is not naturally optically active. The observed optical activity of banded spherulites arises not from the molecular or crystal structure, but rather from the splaying of anisotropic crystalline lamellae in thin films as they twist about the growth direction.^34^ We have recently observed similar behavior in banded spherulites of other achiral molecular semiconductors,^38^ such as tetrathiafulvalene^39,40^ and 2,5-bis(3-dodecyl-2-thienyl)-thiazolo[5,4-*d*] thiazole.^41^ The spherulite sends as few as 4 and as many as 10 angular domains that are alternately left- and right-handed.

Mesoscale twisting is common in melts crystallizing far from equilibrium.^10,42^ For polymers, twisting is associated with the imbalanced stresses arising on polymer lamellae folding surfaces. This process was reviewed recently^43^ and was supported by recent force field calculations.^44^ Intrinsic strain associated with incompatibility between the optimal local packing and 3D crystal periodicity (geometrical frustration) seems to be a reasonable explanation for mesoscale small molecule crystals;^45−47^ however, so far, there is no evidence for its action in TIPS-ADT crystals.

Figure 3b displays the MMI images of the same spherulite in Figure 3a after thermal annealing. The LE signal of the annealed *P*_90_ film coarsens during annealing, reflecting the recrystallization of Form IV into larger Form II crystals. *P*_90_ in-plane orientations were mostly preserved, with a radial distribution of crystal orientations about the spherulite center still evident in the LE_angle_ map. Furthermore, the oscillations in the LE and LE_angle_ maps are weakly preserved in Figure 3b. On the other hand, crystal twisting was not preserved through the recrystallization process, with a complete loss in distinct dextrorotatory and levorotatory domains in the CE map of the annealed *P*_90_ film.

The MMI images collected on the *P*_8_ film before annealing (Figure 3c) exhibit the same features as those collected on the *P*_90_ film, except with a higher oscillation frequency in the LE signal along the radial direction, reflecting the smaller pitch. Coarsening was also observed in the LE map of the *P*_8_ film after thermal annealing (Figure 3d) due to the nucleation and growth of relatively large Form II crystals. The transformed films in Figure 3d show radially elongated needles of Form II, but the crystallographic orientation of these needles is most likely plural since the radial configuration is not evident in the LE_angle_ map in Figure 3d. Systematic oscillations along the radial direction were not observed in the LE and LE_angle_ maps of the annealed *P*_8_ film. The CE map of annealed *P*_8_ films also did not exhibit distinct optically heterochiral domains. Recrystallization was accompanied by the loss of a systematic sense of splay. One can also see in Figure 3a that there is a sign change in the CE signal along the radial direction, which is not an uncommon feature in twisted crystals that has been analyzed previously.^32^ Phenomenologically, this feature is subordinate to the heterochiral sectoring in Figure 3c.

Polycrystalline samples can suffer from depolarization that complicates the differential decomposition in Mueller polarimetry.^48^ A depolarization index (DI) was calculated for the samples in Figure 3. The DI is the Euclidean distance of the normalized Mueller matrix from an ideal depolarizer.^49^ It varies from 0 for an ideal depolarizer to 1. The greater depolarization was found in the *P*_90_ sample with the larger pitch and larger crystallites, as to be expected. The values for unannealed *P*_90_ and *P*_8_ were about 0.5 and 0.8, indicating that the latter is less equivocal in the decomposition and that the heterochirality in the CE image is genuine.

### Crystal Structure Determination

**Figure 4:**
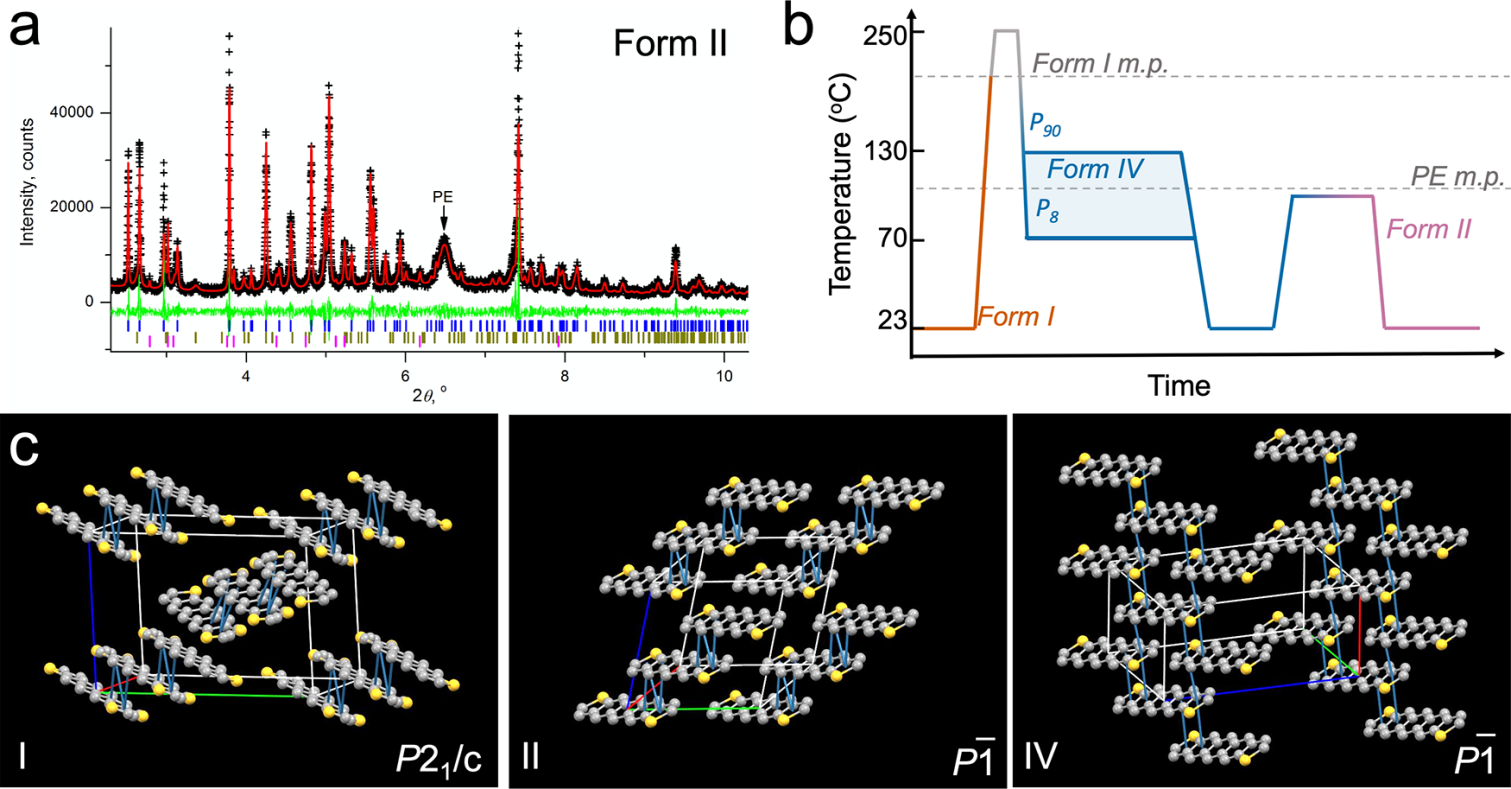
(a) Rietveld refinement of high-resolution synchrotron powder diffraction data collected at 100 K for TIPS ADT Form II containing 16 wt % PE, λ = 0.459722 Å. Observed and calculated intensities are labeled as black crosses and red lines, respectively. Green traces are the difference curves. Reflection positions: blue ticks, Form II; brown tick, Form I; magenta ticks, unknown phase. Broad peak in (a) around 6.5° – PE. (b) Temperature profile to access Forms I, II, and IV. (c) Molecular packing of the four known TIPS ADT polymorphs, with close aromatic interactions visualized by blue lines generated using Aromatics Analyzer 2 in Mercury. Triisopropylsilylethynyl groups and hydrogen atoms are removed for clarity. Form III is illustrated in Figure S10.

After attempts to grow a single crystal of Form II from the melt failed, high-resolution powder X-ray diffraction (PXRD) was performed. High-quality PXRD data set was collected at 100 K at the Advanced Photon Source at Argonne National Laboratory (Figure 4a), but all attempts to index this pattern were unsuccessful. Instead, a film prepared between glass slides and consisting of Form II crystals was carefully heated to 205–210 °C to obtain only a few crystals that were slowly grown by decreasing the temperature stepwise to 195 °C. A single crystal obtained (*ca*. 0.002 × 0.19 × 0.95 mm) was mounted on a GADDS microdiffractometer equipped with a 2D detector. About 35 strong reflections were collected, their corresponding diffraction vectors were determined,^50^ and a triclinic unit cell was fitted. This cell was applied to the high-resolution PXRD pattern and refined using Pawley fit implemented in Bruker TOPAS 6 software with *R*_wp_ = 10.9% (Table 1). The sample contained ∼7% of Form I and was contaminated by some unknown phase. The crystal structure of Form II was solved in the *P*1̅ space group by the simulated annealing procedure implemented in TOPAS 6 software.^51^ A rigid body was constructed based on a TIPS ADT molecule from the experimental Form I (CSD refcode FANGUX) with silyl and all isopropyl groups able to rotate freely. The final refinement provided *R*_p_ = 11.5%, *R*_wp_ = 14.6%, *R*_exp_ = 1.5%, gof = 9.70 (CCDC refcode 2331113). The flipped orientation of anthradithiophene core (Scheme 1a) was introduced, and its occupancy was refined to 34%.

**Table 1:** Crystal Structure Parameters for TIPS ADT Polymorphs Collected at 90 K (Form I) and 100 K (All Other Forms)

	Form I^17^	Form II	Form III	Form IV^18^
*a* (Å)	8.7965(3)	8.5096(3)	7.4587(5)	7.5267(4)
*b* (Å)	17.6967(7)	10.6825(3)	14.9963(8)	7.8596(5)
*c* (Å)	12.1065(5)	11.1556(3)	19.1636(12)	16.6764(8)
α (°)	90.00	69.793(2)	90.00	78.439(9)
β (°)	90.4638(18)	88.392(4)	118.832(7)	89.914(4)
γ (°)	90.00	81.132(3)	90.00	77.249(9)
*V*_unit cell_ (Å^3^)	1882.4	939.947	1877.79	936.083
*V*_unit cell_ (Å^3^)	941.2	939.947	938.895	936.083
*Z*	2	1	2	1
space group	*P*2_1_/*c*	*P*1̅	*P*2_1_/*a*	*P*1̅
melting point (°C)	210.5(5)	207.4(1)	no data	207.7(5)

Another unreported TIPS ADT polymorph was also discovered, hereafter referred to as Form III. Form III spontaneously converted from Form IV in the absence of PE inside a capillary tube during or after transport in dry ice to the Advanced Photon Source. It is also possible that some of it nucleated from the melt during preparation of Form IV. The diffraction pattern was collected at 100 K. The lattice constants were determined with the indexing software McMaille v3.04^52^ and refined using Pawley fit implemented in Bruker TOPAS 6 software^51^ with *R*_wp_ = 12.3% (Figure S10 and Table 1). The PXRD pattern of Form III contained a significant fraction of unconverted Form IV, which was simulated using a Pawley fit obtained for PXRD pattern of uncontaminated Form IV.^8^ The Form III crystal structure was solved in the *P*2_1_/*a* space group by the same procedure described for Form II (Figure S10 and Table 1) and refined with the flipped orientation of anthradithiophene core 34% and final *R*_p_ = 14.7%, *R*_wp_ = 18.4%, *R*_exp_ = 4.3%, gof = 4.25 (CCDC refcode 2331112). Relatively high values of *R*-factors for Forms II and III are likely related to orientational disorder in silyl groups, which is found for Form I (CCDC refcode FANGUX) and manifests itself by variations in half-widths of reflections with similar 2θ positions. Multiple attempts to isolate Form III in films were unsuccessful.

Figure 4b displays the temperature profile followed to access Forms I, II, and IV. Form II was only observed when PE was present in the film. Figure 4c displays the molecular packing for TIPS ADT Forms I, II, and IV. As identified with Aromatics Analyzer 2 in Mercury,^53^ Forms I and II exhibit one-dimensional chains of close interactions while Form IV exhibits a two-dimensional network of interactions consistent with “offset stack” intermolecular aromatic units.

### Polymorph-Dependent Optoelectronic Properties

**Figure 5:**
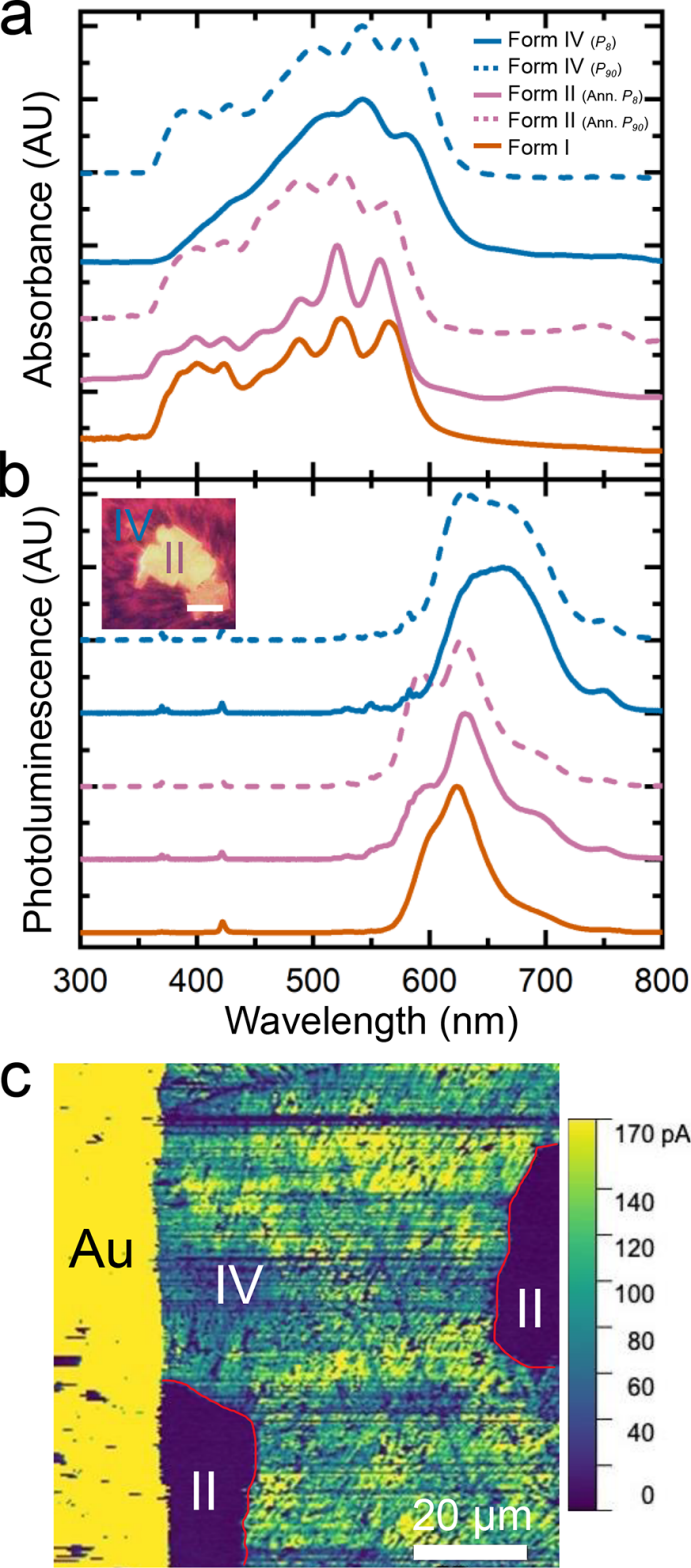
(a) Absorbance spectra and (b) photoluminescence spectra (λ_ex_ = 436 nm) collected on Forms I, II, and IV. Spectra for *P*_8_ and *P*_90_ films are provided. Inset in (b) displays a fluorescence micrograph on a partially annealed TIPS ADT film with both Forms IV and II present. (c) Conductive AFM current map collected on a partially annealed *P*_8_ TIPS ADT film with both Forms IV and II exhibiting brickwork and slipstack packing motifs, respectively. Form II regions highlighted in red.

Optoelectronic properties in acene derivatives traditionally have been inferred by comparing compounds that exhibit either slipstack or brickwork packing in the room-temperature thermodynamically stable state.^9^ Here, we can compare directly the two structures within the same compound, eliminating any differences due to molecule-specific electronic structures. Figure 5a displays the solid-state absorption spectra of Forms I, II, and IV. Forms I and II exhibit the same transitions in the absorption spectra at 488, 524, and 565 nm. These transitions are consistent with previously published spectra of Form I.^8^ In comparison, the Form IV absorption spectrum is red-shifted, with main peaks at 502, 542, and 579 nm (see Figure S11 for the polarization angle-dependent absorption spectra). Bathochromic shifts in Form IV reflect an increased π overlap in its 2D brickwork J-aggregate-like network compared to that in the 1D network of Forms I and II.^54^

Figure 5b contains the normalized emission spectra of each polymorph (λ_ex_ = 436 nm). Photoluminescence from all three polymorphs is most intense at 626 nm. Form IV exhibits an additional peak at 667 nm, while Form II shows a second peak at 593 nm. Forms I and II are more emissive than Form IV (Figure 5b inset), consistent with less extensive π networks. Molecular packing at the angstrom length scale and film microstructure at the submicron to millimeter length scale both impact overall organic electronic device performance.^55^ Conductive atomic force microscopy (c-AFM) maps were collected on partially annealed TIPS ADT films to directly compare the conductivities of polycrystalline Form IV banded spherulites and Form II single crystals. Lateral conductivity through a partially annealed *P*_8_ TIPS ADT film was mapped by evaporating a gold electrode on the film surface and applying a 10 V bias across the electrode and conductive AFM tip while scanning the film surface. Figure 5c displays the AFM current map of a region scanned across the edge of the gold electrode and TIPS ADT film with both Form IV and Form II regions present (see Figure S12 for the corresponding height map). Current levels in the Form IV banded spherulite region oscillated between 80 and 170 pA due to different charge injection and extraction efficiencies along the ⟨001⟩ and ⟨100⟩ directions in alternating bands.^8^ Current levels through Form II single crystals, on the other hand, were near 0 pA even though these crystals are free of high-resistivity grain boundaries.^56,57^ The current level was low for both the Form II crystal in contact with the gold electrode and the Form II crystal to the right of the electrode, indicating that polymorph-dependent interactions with the gold contact is not the primary factor affecting current flow. Instead, we expect that this large difference in conductivities between Form IV and Form II are a consequence of the more extensive π network in Form IV compared to Form II.

## Conclusions

Here, we report a postprocessing thermal annealing method to tune polymorphism, molecular orientation, and microstructure in thin films consisting of TIPS ADT with 16 wt % PE. Recrystallization of Form IV to Form II was enabled by enhanced PE mobility, which in turn increased the mobility of TIPS ADT molecules. This polymorph transition was not observed in TIPS ADT-only films, suggesting the incorporation of polymer additives as a viable strategy for polymorph discovery. Optoelectronic characterization of partially annealed TIPS ADT films highlights the importance of polymorphism in dictating material properties—even though Form II formed as single crystals without grain boundaries that act as barriers to charge transport, its electrical conductivity was significantly lower than polycrystalline films of Form IV. Form II, on the other hand, is more emissive than Form IV. Polymer-aided discovery of molecular polymorphs should inform structure–function relationships in organic semiconductors while providing a method of tuning optoelectronic properties.

## Experimental Methods

### Materials

TIPS ADT was synthesized according to a previously published procedure.^58^ Low density PE (0.925 g/mL at 25 °C), medium density PE (0.94 g/mL at 25 °C), and high density PE (0.952 g/mL at 25 °C) were purchased from Sigma-Aldrich and used without further purification. TIPS ADT and PE powders were mixed in a 5:1 weight ratio (corresponding to 16 wt % PE) and ground together with a lab-grade mortar and pestle. TIPS ADT-PE thin films were fabricated by placing 1–2 mg of TIPS ADT-PE powder between two glass slides, melted on a Kofler bench at 250 °C, then rapidly cooled for 10 s at *T*_c_ = 70 °C or *T*_c_ = 130 °C depending on the desired twisting pitch.

### Thermal Annealing

TIPS ADT-PE films were thermally annealed at 100 °C for 60 min in a Mettler FP82HT Hot Stage to induce complete recrystallization. Films were first inserted into the hot stage, after which the temperature was ramped from room temperature to 100 °C at a rate of 20 °C/min. At the 60 min mark, films were immediately removed from the hot stage and cooled to room temperature on the lab bench.

### Optical Characterization

Optical micrographs were collected with an Olympus BX53 microscope fitted with crossed polarizers. Timelapse videos of optical micrographs were created in ImageJ, and frames were stabilized with the fixTranslation macro plugin.^59^ MM images were collected on a home-built instrument consisting of a cross-polarizer microscope with two rotating quarter-wave plates above and below the sample, which form a complete polarization state generator and polarization state analyzer, in accordance with previously published designs.^29,31,60−62^ Local absorbance and PL spectra were obtained with a CRAIC Technologies 508 PV microspectrophotometer. PL spectra were taken with an excitation wavelength of 436 nm.

### Linear Growth Rate Kinetics

*P*_8_ films were continuously thermally annealed at desired temperatures from 170 to 50 °C using a Mettler FP82HT Hot Stage to induce recrystallization. Films were first inserted into the hot stage at a given temperature. After temperature stabilization for 30 s, the lengths of five Form II crystals from different areas of the film were measured along and perpendicular to the long axis of the crystals from time-lapsed images from the Olympus BX53 microscope. The temperature was then ramped to a different temperature at a rate of 20 °C/min, and this procedure was repeated. Data in Figure 1b correspond to the measurements on three different films.

### X-ray Diffraction

2D XRD patterns were collected on TIPS ADT films, thin single crystals, and powders with a Bruker D8 Discover General Area Detector Diffraction System equipped with a VÅNTEC-2000 2D detector and Cu-*K*α source (λ = 1.54178 Å). The X-ray beam was monochromated with a graphite crystal and collimated with a 0.5 mm capillary collimator (MONOCAP).

### High-Resolution X-ray Powder Diffraction

High-resolution synchrotron PXRD data were collected at the 11-BM beamline (Advanced Photon Source, APS, at Argonne National Laboratory) using an average wavelength of λ = 0.459722 Å (Form II) and λ = 0.458955 Å (Form III). All measurements were performed at *T* = 100 K. The sample of Form II was prepared by melting powder of TIPS ADT/PE 5:1 mixture in 0.8 mm Kapton capillary at 250 °C and cooling it to room temperature within seconds. Then, the capillary was annealed at 100 °C for 30 min and measured immediately after that. For the data collection, discrete detectors covering a 26° angular range from −2 to 2° 2θ were scanned every 0.001° 2θ at 0.002°/s. The final data set combined 15 scans over the 2θ range from 0.5 to 24° and totaling 510 min. To get Form III, TIPS ADT powder was melted in 0.8 mm Kapton capillary at 250 °C and cooling it to room temperature within seconds. Then, the absence of preferred orientations was confirmed by collecting a PXRD pattern in transmission mode using a Bruker D8 GADDS system. The sample was placed into a box with dry ice and shipped to APS. For the data collection, discrete detectors covering a 34° angle range from −6 to 16° 2θ with data points every 0.001° 2θ and a scan speed of 0.01°/s. The pattern contained data from 0.5 to 30° and was collected within 30 min.

### Differential Scanning Calorimetry (DSC)

DSC measurements were conducted under an inert N_2_ atmosphere at a scan rate of 10 °C/min with a PerkinElmer DSC 8000 equipped with an Intracooler 2. For the pure TIPS ADT, low density PE, medium density PE, and high density PE measurements, powder was loaded as is into the standard Mettler aluminum crucibles. For the film measurements, eight films of each type were first crystallized as described previously. Then, the glass slides were separated, and the crystallized material was neutralized with an electrostatic gun before it was scraped off and loaded into the DSC crucibles. Each DSC sample weighed around 1.5 mg.

### Scanning Electron Microscopy

Films were crystallized at temperatures indicated previously on a gold-coated silica substrate. After crystallization, the silica cover slide was removed, leaving gold on the film surface. Films were then cut at the desired cross section and imaged. Cross-section edges were immersed in acetone for ∼1 min to selectively dissolve TIPS ADT, preserving the PE texture. The samples were sputter-coated with ∼2 nm of iridium prior to imaging. Cross-sectional scanning electron micrographs were collected with a Merlin field-emission scanning electron microscope (Carl Zeiss) using an in-lens detector, with an electron high tension voltage of 4.00 kV and current of 110 pA.

### Atomic Force Microscopy

*P*_*8*_ films were crystallized as previously described but with a fluorinated self-assembled monolayer (FSAM) coated cover slide that was easily removed. These films were thermally annealed for 30 min to induce partial recrystallization, after which the FSAM cover slide was removed. Gold electrodes were deposited via thermal evaporation near film regions of interest for current measurements.

## Supporting Information Available

Brightfield optical micrographs, differential scanning calorimetry curves, optical micrographs of annealed TIPS ADT-only film, top-down SEM images of annealed films, *in situ* powder X-ray diffraction patterns during annealing, time lapse videos of films during annealing. (PDF)
Frame-by-frame polarized optical micrographs of the annealing and recrystallization process in a *P**_8_* film (AVI)
Frame-by-frame polarized optical micrographs of the annealing and recrystallization process in a *P_90_* film (AVI)
Crystallographic data of TIPS ADT Form II (CIF)
Crytallographic data of TIPS ADT Form III (CIF)

## Supplementary Material

cm4c00418_si_001.pdf

cm4c00418_si_002.avi

cm4c00418_si_003.avi

cm4c00418_si_004.cif

cm4c00418_si_005.cif
