# Supplementary material for: Polymer-Assisted Polymorph Transition in Melt-Processed Molecular Semiconductor Crystals
Source: Chem Mater. 2024 Jun 3;36(12):5976–85. doi: 10.1021/acs.chemmater.4c00418 (PMC11209941; doi:10.1021/acs.chemmater.4c00418)
Supplement: Supplementary file 1 — cm4c00418_si_001.pdf [file cm4c00418_si_001.pdf]

Supplementary Information for

Polymer-assisted polymorph transition in melt-processed molecular semiconductor crystals

Pallavi Sundaram,[a,†] Rochelle B. Spencer,[a,†] Akash Tiwari,[a] St. John Whittaker,[a] Trinanjana Mandal,[a] Yongfan Yang,[a] Emma K. Holland,[b] Christopher J. Kingsbury,[c] Mia Klopfenstein,[a] John E. Anthony,[b] Bart Kahr,[a] Sehee Jeong,[a,]* Alexander G. Shtukenberg,[a,]* Stephanie S. Lee[a,]*

[a]Molecular Design Institute, Department of Chemistry, New York University, New York, NY 10003, USA

[b]Department of Chemistry, University of Kentucky, Lexington, Kentucky 40506, USA

[c]Cambridge Crystallographic Data Centre (CCDC), 12 Union Road, Cambridge CB2 1EZ, UK

*sj3685@nyu.edu, as5243@nyu.edu, stephlee@nyu.edu

[†]these authors contributed equally

Video S1. Frame-by-frame polarized optical micrographs of the annealing and recrystallization process in a P_8 film. Timelapse videos of optical micrographs were created in ImageJ and frames were stabilized with the fixTranslation macro plugin.

Video S2. Frame-by-frame polarized optical micrographs of the annealing and recrystallization process in a P_{90} film. Timelapse videos of optical micrographs were created in ImageJ and frames were stabilized with the fixTranslation macro plugin.

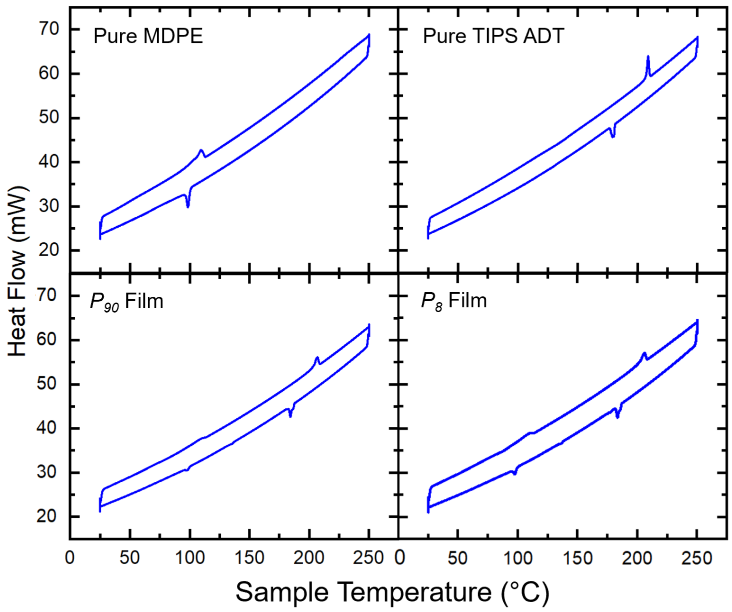

Figure S1. Differential scanning calorimetry (DSC) curves for (a) pure medium density PE, (b) pure TIPS ADT, (c) P_{90} film material, and (d) P_8 film material. P_{90} film material was scraped off glass substrates holding TIPS ADT/MDPE films crystallized from the melt at 130 °C. Similarly, P_8 film material was scraped off glass substrates held TIPS ADT/MDPE films crystallized from the melt at 70 °C. DSC measurements were conducted under an inert N_2 atmosphere at a scan rate of 10 °C/min with a Perkin Elmer DSC 8000 equipped with Intracooler 2. DSC samples all weighed around 1.5 mg.

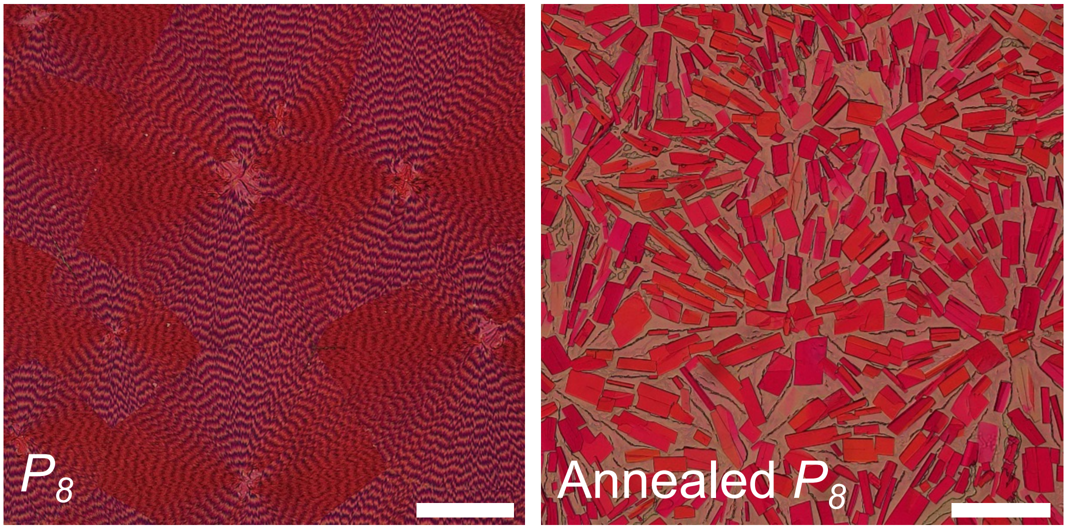

Figure S2. Brightfield optical micrographs of a TIPS ADT/MDPE film grown from the melt with a crystallization temperature of 70 °C (P_8) before (left) and after (right) thermally induced recrystallization. Scale bar = 100 µm.

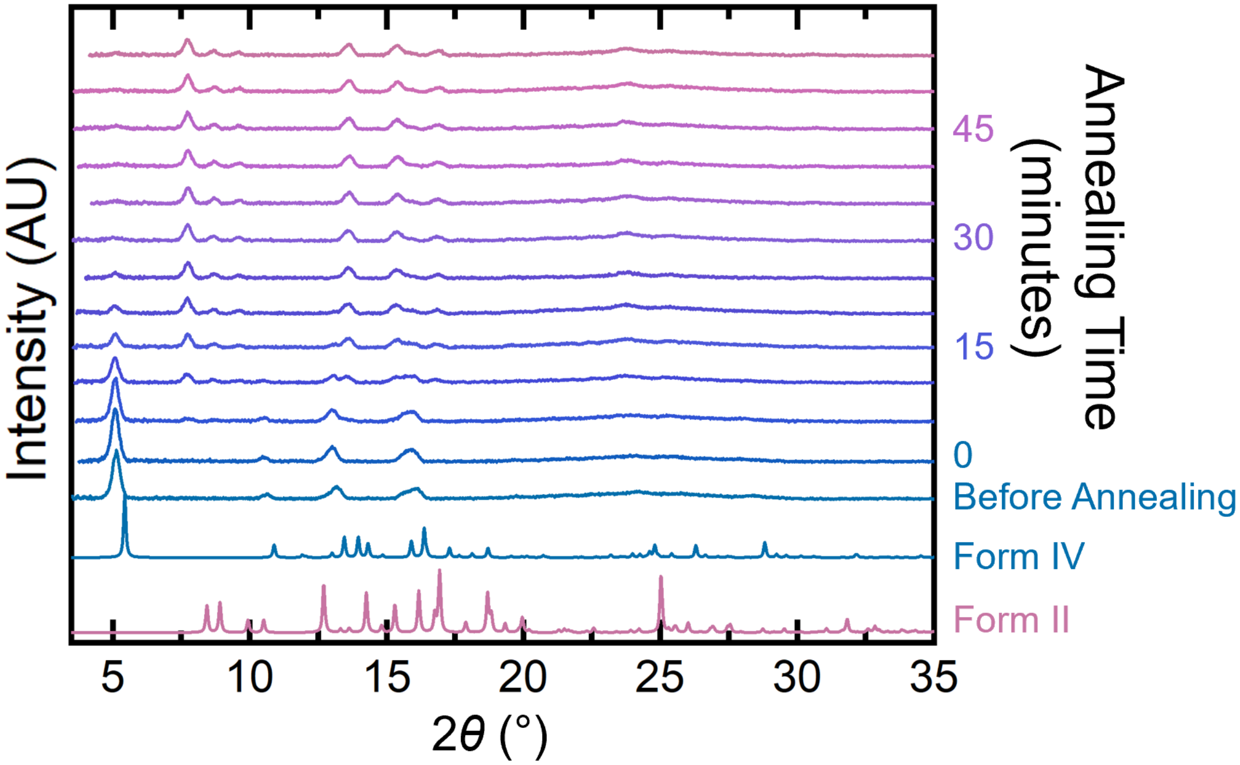

Figure S3. Powder XRD patterns of a P_8 film (TIPS ADT/MDPE film crystallized from the melt at 70 °C) during one hour of *in situ* thermal annealing at 100 °C. PXRD patterns were collected simultaneously with thermal annealing. Simulated powder patterns from the single crystal structures of Form II and IV are provided at the bottom. Systematic shifts in peaks between the single crystal pattern collected at -196 ºC and the film patterns collected at 100 ºC are likely due to thermal expansion of the crystal lattice.

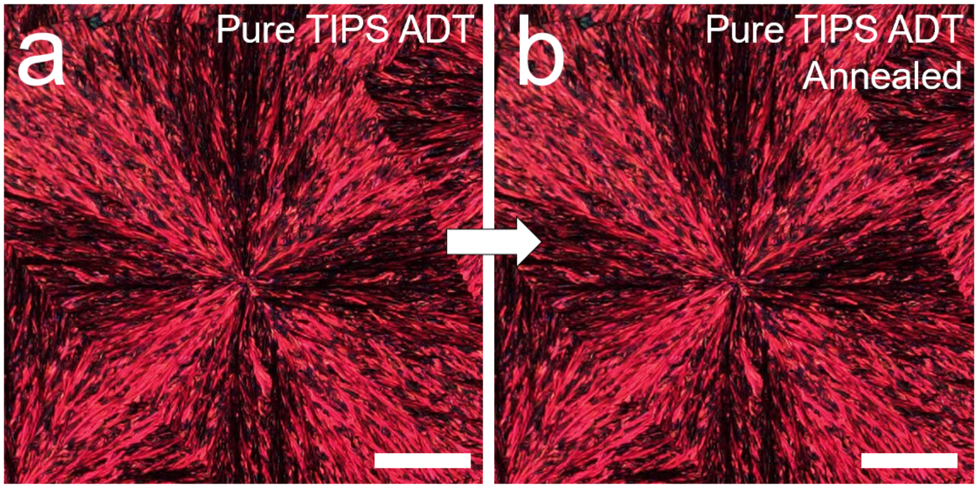

Figure S4. Optical micrographs of a pure TIPS ADT film between crossed polarizers grown from the melt with a crystallization temperature of 130°, before (left) and after (right) thermal annealing. Scale bars = 100 µm.

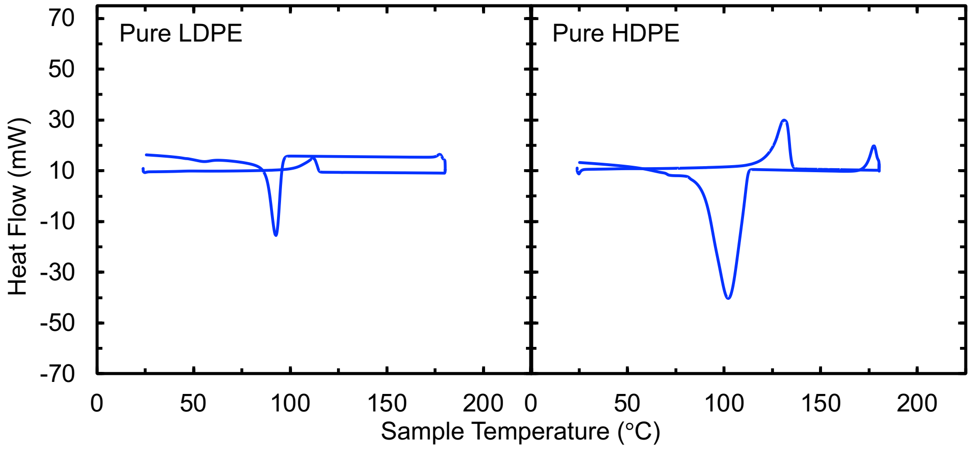

Figure S5. DSC curves for pure low density PE (LDPE) and high density PE (HDPE).

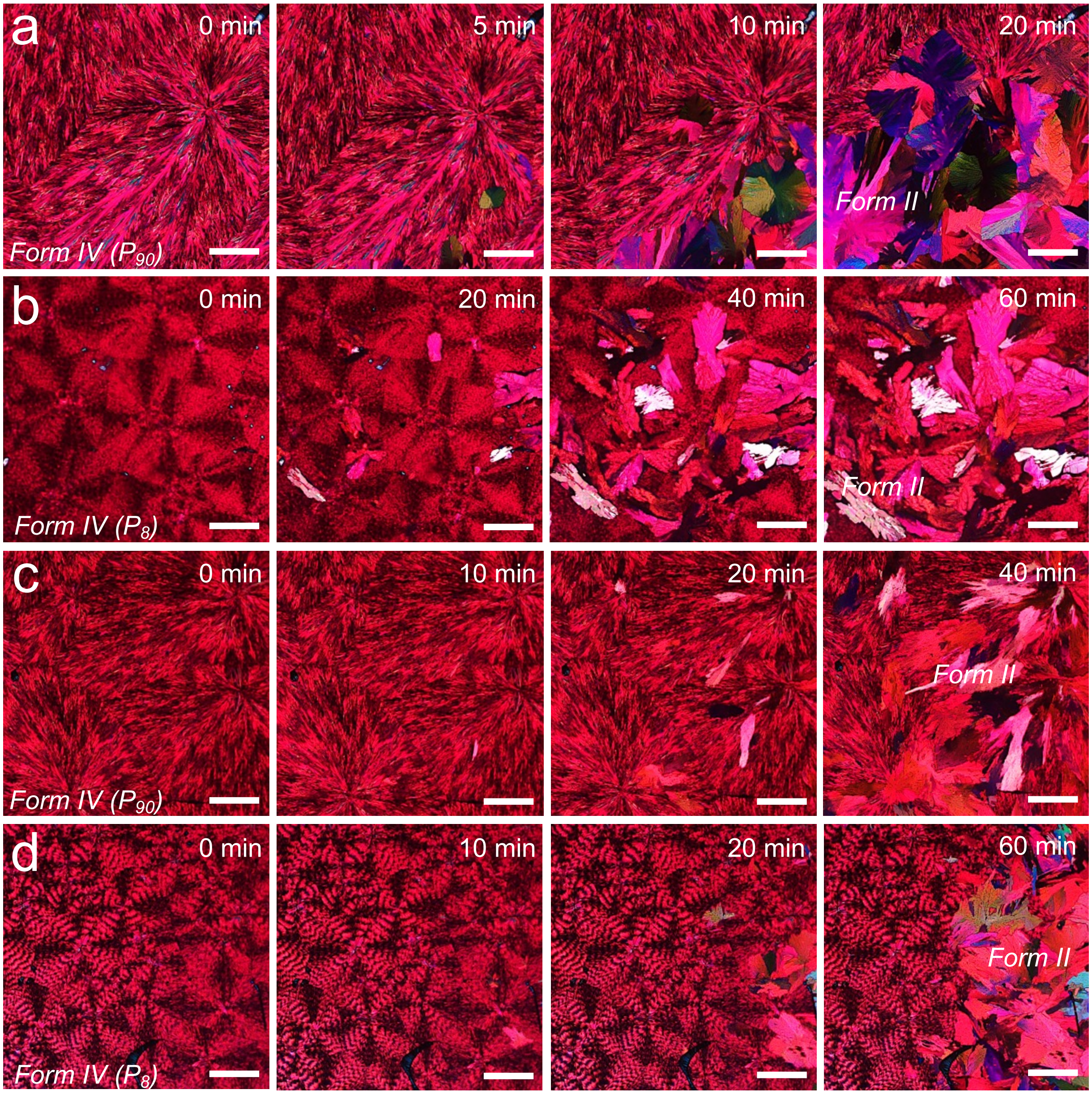

Figure S6. Time-dependent cross-polarized optical micrographs of TIPS ADT/LDPE (a) P_{90} and (b) P_8 films and TIPS ADT/HDPE (c) P_{90} and (d) P_8 films taken *in situ* during thermal annealing at 100 °C. Scale bar = 100 μm.

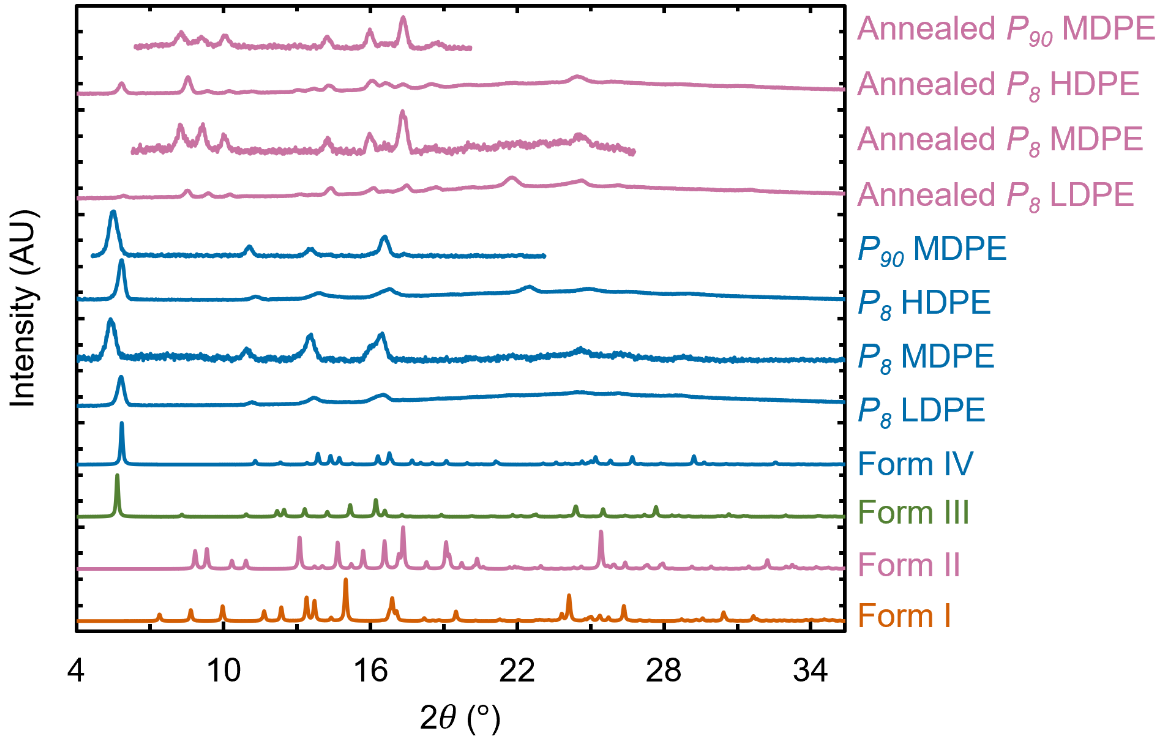

Figure S7. Powder XRD patterns collected on TIPS ADT/PE films crystallized from the melt at 70 °C (P_8) and 130°C (P_{90}), with different PE densities—low density (LD), medium density (MD), and high density (HD). Powder XRD data shown was collected on films both before and after annealing for 60 min at 100 °C. Forms I, II, III and IV powder patterns (bottom) are simulated patterns generated from the molecular packing structure using Mercury.

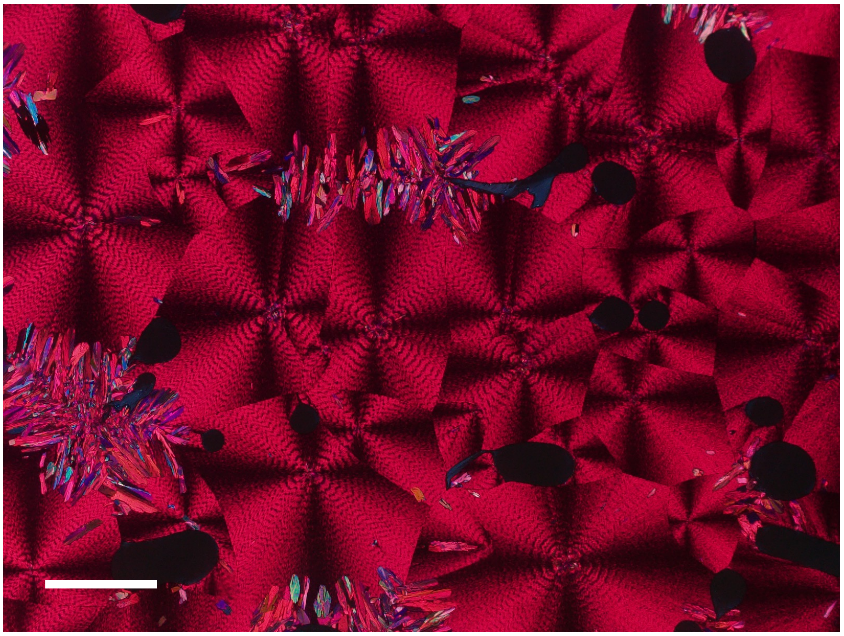

Figure S8. Representative optical micrograph collected between crossed polarizers of a TIPS ADT Form IV film partially converted to Form II at 85 °C. Scale bar = 200 µm.

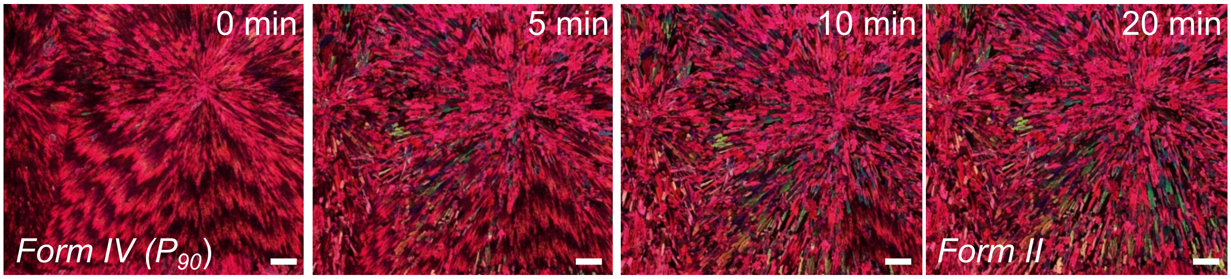

Figure S9. Time-dependent cross-polarized optical micrographs of a TIPS ADT/MDPE P_{90} film taken *in situ* during thermal annealing at 100 °C. See also Video S2 for the timelapse of this transformation. Scale bar = 100 µm.

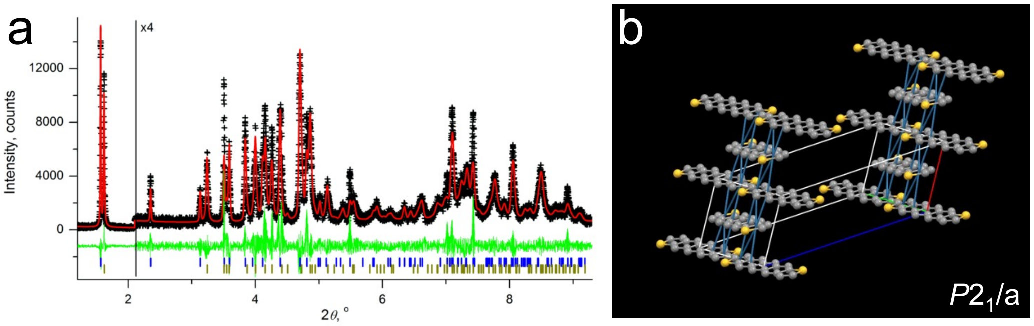

Figure S10. a) Rietveld refinement of high-resolution synchrotron powder diffraction data collected at 100 K for TIPS ADT Form III containing 16 wt.% medium density PE, λ = 0.459722 Å. Observed and calculated intensities are labeled as black crosses and red lines, respectively. Green traces are the difference curves. Reflection positions: blue ticks - Form II, brown tick – Form I. b) Molecular packing of TIPS ADT Form III, with close aromatic interactions visualized by blue lines generated using Aromatics Analyser 2 in Mercury. Triisopropylsilylethynyl groups and hydrogen atoms are removed for clarity.

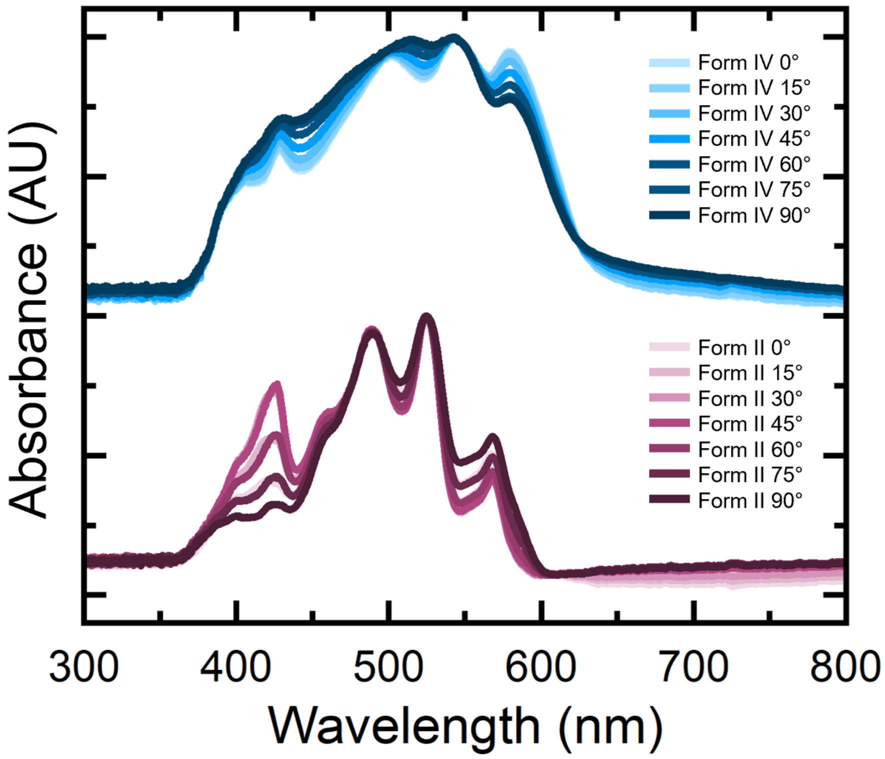

Figure S11. Polarization angle-dependent absorbance spectra for Form IV (top) and Form II (bottom), collected on a P_8 film before and after thermal annealing at 100 °C for 1 hour, corresponding to the respective forms. Polarization angle of the incident light was adjusted at 15° intervals using a linearly polarized filter.

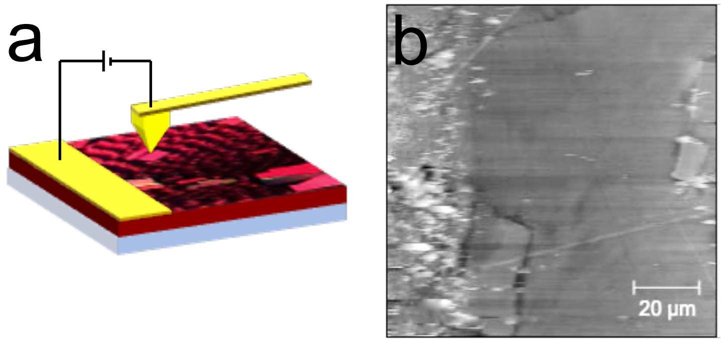

Figure S12. (a) Device geometry to measure lateral current flow through a TIPS ADT film using conductive atomic force microscopy. (b) Height map corresponding to conductive AFM measurement data collected on a partially annealed P_8 film as shown in Fig. 5c.
